# Compensatory Effects of Long-Term Care Insurance on Mental Health Among Rural Older Women: Evidence from China

**DOI:** 10.3390/healthcare14152323

**Published:** 2026-08-01

**Authors:** Zhangbo An, Qihui Xie, Hongying Zhang, Yuwu Xie

**Affiliations:** 1 School of Government, Beijing Normal University, Beijing 100875, China; 2 School of Laws and Humanities, China University of Mining and Technology-Beijing, Beijing 100083, China

**Keywords:** long-term care insurance, gendered institutional compensation, compensatory substitution, mental health, older women

## Abstract

**Background:** Rural older women in China face intersecting disadvantages including eroded family support, limited pension coverage, and high social isolation that elevate depressive symptoms. Although Long-Term Care Insurance (LTCI) has expanded rapidly since 2016 to provide both financial protection and formal care services for older adults, whether it can compensate for depleted informal support and protect mental health among this vulnerable population remains unknown. **Methods:** We used five waves of CHARLS (2011–2020) with 2193 rural women aged ≥60. We employed a staggered difference-in-differences design, complemented by moderation analysis, heterogeneity tests, and time-dependent survival analysis. Robustness checks included Callaway-Sant’Anna DID, placebo permutation and baseline exclusion sensitivity. **Results:** LTCI reduced depression scores by 0.45 points (β = −0.447, *p* = 0.028) and depression onset hazard by 36.1% (HR = 0.639, *p* = 0.009). Benefits were compensatory, concentrated among women lacking regular emotional and financial support from children and those with high social isolation. Heterogeneity analyses demonstrated a pronounced age gradient and a significant education gradient, while care-need and disability subgroups showed directionally consistent but underpowered effects. These findings were robust to supplementary sensitivity checks, including Callaway-Sant’Anna DID, placebo permutation, and baseline-depressed exclusion. **Conclusions:** LTCI confers meaningful mental health protection for rural older women through compensatory pathways that substitute for depleted informal support. However, benefits are concentrated among younger-old and more-educated women, raising equity concerns. Gender-sensitive implementation with mental health screening, targeted outreach for less-educated women, and community-based enrollment assistance is essential to ensure policy equity.

## 1. Introduction

The twenty-first century is characterized by a profound and unprecedented demographic transition toward population aging. Globally, the proportion of individuals aged 60 and above is projected to double by 2050, placing immense pressure on health systems, social security frameworks, and traditional models of intergenerational care [1]. China exemplifies this transformation on an accelerated scale. The population aged 60 and older has surpassed 280 million, nearly one-fifth of the total. The Chinese government has responded with ambitious policy reforms, most notably the staggered rollout of Long-Term Care Insurance (LTCI), a social insurance mechanism that combines economic protection with direct care support for functionally dependent older adults [2,3]. On the financing side, LTCI pools payroll-based contributions (typically 0.5–1% of wages) with government subsidies to reduce out-of-pocket care expenditures and mitigate the financial risks associated with chronic disability and dependency. On the service side, eligible beneficiaries, typically older adults assessed with severe limitations in activities of daily living (ADL) or instrumental activities of daily living (IADL), receive tangible care assistance, including institutional admission, home- and community-based nursing and personal care, and, in some pilots, cash allowances that enable family caregivers to purchase care. Through this dual economic-and-care design, LTCI aims not only to alleviate financial strain but also to substitute for or supplement eroded informal caregiving when family support is unavailable. Implementation began in 2016 with pilot cities including Qingdao, and the program has gradually expanded to additional municipalities. A growing body of research has already evaluated the effects of LTCI on healthcare utilization, subjective well-being, medical expenditure, and financial protection [4,5,6,7,8,9]. However, the psychosocial consequences of this reform and the extent to which mental health gains operate through care provision as well as economic relief, particularly through a gender lens, remain inadequately understood.

The challenges of aging in China are most acute in rural regions, where decades of selective out-migration of younger adults have eroded the traditional family support infrastructure, leaving behind a population that is disproportionately elderly and female [10,11,12]. Rural older women occupy a uniquely precarious position at the intersection of multiple disadvantages. Having often spent their working lives in informal labor, many lack adequate pension coverage or sufficient financial assets for a secure retirement. Simultaneously, the erosion of filial piety and geographic separation have diminished the availability of informal caregiving. Due to deeply entrenched patriarchal gender roles, rural women have historically provided such care and, paradoxically, are now increasingly deprived of receiving it [13]. This confluence of economic scarcity, physical morbidity, and weakened social networks has precipitated a mental health crisis.

Epidemiological evidence consistently documents elevated rates of clinically significant depressive symptoms among rural older women in China, with prevalence estimates substantially higher than those observed among urban or male counterparts [10]. This heightened vulnerability can be conceptualized through the lens of gendered pathways to health, which posits that the distribution of illness is shaped by differential exposure and vulnerability to social, economic, and environmental stressors that accrue over the life course [14]. Social isolation and loneliness have been identified as independent risk factors for late-life depression and mortality [15,16,17], with rural older women particularly susceptible due to the erosion of intergenerational co-residence. Furthermore, ageism, the stereotyping and discrimination directed against older adults, compounds gender disadvantage, as older women face intersecting age- and gender-based biases that limit their access to both formal services and informal support [18]. Despite the gravity of this challenge, the extent to which formal social policies such as LTCI can interrupt these gendered pathways remains a critical empirical gap.

A central theoretical question motivating this study concerns whether formal insurance substitutes for or complements pre-existing informal support structures. The compensatory substitution model predicts that formal LTCI coverage will yield the greatest psychological benefits for individuals whose informal support networks are weakest, by filling the void left by absent or diminished family resources [19,20]. The complementary model, in contrast, predicts that formal care and informal support function synergistically, with individuals who already enjoy robust social ties deriving enhanced benefits from LTCI. Distinguishing between these two pathways carries direct implications for the targeting of LTCI outreach and the equitable distribution of policy benefits across subpopulations with varying levels of social capital.

We hypothesize, based on the theoretical primacy of resource substitution in contexts of structural disadvantage [14], that LTCI will exhibit a compensatory pattern, conferring the most pronounced mental health benefits upon rural older women who lack regular emotional contact with their children, who receive no financial transfers, and who experience high levels of social isolation.

This study is expected to make several distinct contributions. By focusing exclusively on rural older women, we move beyond gender-blind evaluations and examine LTCI as institutional compensation at the intersection of patriarchal care expectations and rural deprivation. We investigate moderating roles of emotional and financial support, social isolation, and pension coverage to clarify whether LTCI substitutes for or amplifies informal resources. A staggered difference-in-differences design reinforced by Callaway-Sant’Anna DID, placebo tests, and time-dependent survival analysis provides robust evidence on both depressive symptom severity and depression onset. Subgroup analyses by age, education, and functional status examine whether benefits are equitably distributed.

## 2. Methods

### 2.1. Data Source and Sample Construction

This study utilized data from the China Health and Retirement Longitudinal Study (CHARLS), a nationally representative longitudinal survey of adults aged 45 and older and their spouses [21]. We employed five waves in 2011, 2013, 2015, 2018, and 2020. The baseline wave in 2011 established the pre-intervention health and socioeconomic profiles of respondents, while subsequent waves captured the dynamic changes following the staggered introduction of LTCI across pilot cities beginning in 2016 [3]. CHARLS employs a multistage stratified probability proportional-to-size sampling design, covering 28 provinces, 150 counties, and 450 villages/urban communities across China. Data are collected through face-to-face computer-assisted personal interviews (CAPI) conducted in respondents’ homes, supplemented by physical measurements and blood samples. The survey achieves a high response rate (over 80% at baseline) and has been widely used in aging and health research [21].

Our analytical sample was constructed through sequential exclusions from the full female CHARLS sample. We retained women who resided in rural areas at baseline, were aged 60 or older at baseline, and had non-missing observations on the CES-D scale and core covariates. Respondents who migrated across urban and rural boundaries during the observation period were excluded to avoid confounding LTCI effects with changing residential environments. The final sample comprised 2193 rural older women contributing 8803 person-wave observations, of whom 572 resided in cities that implemented LTCI pilot programs during the study period. The treatment (case) group comprised women residing in cities that implemented LTCI pilot programs during the observation period (*n* = 572), while the control group comprised women in cities without LTCI implementation throughout the study (*n* = 1621). Treatment assignment was based on city-level policy implementation status, not individual enrollment.

### 2.2. Variable Definitions

The primary outcome variable was depressive symptoms measured by the Center for Epidemiologic Studies Depression Scale (CES-D), a widely validated 10-item instrument that assesses the frequency of depressive symptoms over the past week [22]. Each item is scored from 0 to 3, yielding a total score ranging from 0 to 30, with higher scores indicating more severe symptoms. A cutoff of 10 or above identified clinically significant depressive symptoms [23]. The CES-D has demonstrated satisfactory reliability and validity in Chinese older adult populations, with Cronbach’s alpha typically exceeding 0.80 [24].

The core explanatory variable was LTCI exposure, coded as 1 if the respondent resided in a city that had implemented an LTCI pilot program by the survey year and 0 otherwise. Pilot city status was verified through official documents issued by the Ministry of Human Resources and Social Security and city-level policy announcements.

Moderation variables captured distinct dimensions of pension coverage, family support and social integration. Emotional support was measured as a binary indicator of whether the respondent had face-to-face contact with children at least once per week. Financial support was a binary indicator of whether the respondent received any financial assistance from children during the past year [13,25]. Social isolation was measured using a composite index ranging from 0 to 6, constructed from items assessing the frequency of social activities, community participation, and contact with non-family social networks, with higher scores indicating greater social isolation [26]. Pension coverage was defined as a binary indicator of whether the respondent received any public pension income.

Control variables included age in years, marital status (married or partnered versus unmarried, widowed, or divorced), activities of daily living limitations (count of difficulties across six ADL items), chronic disease count (number of diagnosed conditions), and educational attainment (classified into four categories from illiterate to middle school and above [27]).

### 2.3. Empirical Strategy for Main Effects

#### 2.3.1. Two-Way Fixed Effects and Goodman-Bacon Decomposition

Our primary causal identification strategy is a staggered difference-in-differences design exploiting the timing of LTCI pilot rollout across cities. Supplementary estimators including Callaway-Sant’Anna DID, placebo permutation, baseline-depressed exclusion, and time-dependent survival models are used to assess robustness.

To estimate the causal effect of LTCI on depressive symptoms, we employed a multi-period difference-in-differences (DID) model that exploits the staggered timing of LTCI implementation across pilot cities [28,29,30]. As a benchmark, we first estimate a two-way fixed effects (TWFE) model of the form:

(1) $$Y_{it}=\beta_{0}+\beta_{1}\cdot LTCI_{ct}+\beta_{2}\cdot X_{it}+\mu_{i}+\lambda_{t}+\varepsilon_{it}$$

where $Y_{it}$ denotes the CES-D score for individual i at time t; $LTCI_{ct}$ is the binary treatment indicator for city c at time t; $X_{it}$ represents a vector of time-varying individual-level covariates including age, marital status, ADL limitations, and chronic disease count; $\mu_{i}\;$ captures individual fixed effects that absorb time-invariant unobserved heterogeneity. $\lambda_{t}$ accounts for unobserved time-invariant heterogeneity and common time trends and $\varepsilon_{it}$ is the idiosyncratic error term. The coefficient β represents the DID estimator of interest, capturing the average treatment effect of LTCI on CES-D scores under the parallel trend assumption [31]. Standard errors are calculated using the Driscoll-Kraay method to accommodate cross-sectional and serial correlation at the city level [32], with additional inference based on 500 bootstrap replications.

Under staggered adoption, however, the TWFE estimator can be biased due to negative weighting (Goodman-Bacon, 2021) [33]. We therefore conduct a Goodman-Bacon decomposition of the TWFE model, which partitions the overall estimate into a weighted average of three types of 2 × 2 comparisons: (1) treated vs. never-treated; (2) early-treated vs. later-treated (using the later-treated as controls before their own treatment); and (3) later-treated vs. early-treated (using already-treated units as controls). The third type receives negative weights and constitutes the primary source of bias.

#### 2.3.2. Moderation Analysis

To examine the moderating roles of emotional support, financial support, and social isolation, we extended the baseline model by including interaction terms between LTCI and each moderator:

(2) $$Y_{it}=\alpha +\beta_{1}\cdot LTCI_{ct}+\beta_{2}\cdot Moderator_{it}+\beta_{3}\cdot \left(LTCI_{ct}\times Moderator_{it}\right)+\gamma \cdot X_{it}+\mu_{i}+\lambda_{t}+\varepsilon_{it}$$

The coefficient $\beta_{3}$ captures the differential effect of LTCI across levels of the moderator variable, revealing whether the mental health benefits of LTCI are conditioned by family support structures and social integration [34].

#### 2.3.3. Subgroup Analysis

For subgroup analysis, we stratified the sample by age group, care need (binary), disability status (binary), and educational attainment (binary), reflecting the empirical distribution of the sample, to examine whether LTCI effects differed according to human capital endowments that may shape health literacy, information access, and ability to navigate the LTCI application process [35].

Educational attainment, described as a four-category variable in Section 2.2, was collapsed into a binary indicator (educated versus illiterate) for the heterogeneity analysis. This decision was motivated by the highly skewed distribution of education in the sample (mean = 1.21, indicating the vast majority of rural older women are illiterate or have only informal literacy education). The resulting small cell sizes in the higher education categories would produce unstable estimates with insufficient statistical power. The binary classification preserves the key theoretical distinction between women with any formal schooling and those without that is most relevant to health literacy and administrative navigation capacity.

#### 2.3.4. Survival Analysis

To complement the primary analysis of continuous CES-D score changes, we employed survival analysis to estimate the effect of LTCI on the hazard of new-onset depression. Because LTCI exposure is time-varying, individuals are unexposed until the year their city implements the pilot program, treating LTCI as a baseline fixed covariate in a conventional Cox model would introduce immortal time bias (i.e., person-time before LTCI adoption would be incorrectly classified as “treated”). We therefore used a time-dependent Cox proportional hazards model, which allows the exposure status to vary over time within the same individual [36,37].

To further tighten the control for age as the dominant risk factor for late-life depression, we adopted age as the time scale. Accordingly, the model handles left truncation, respondents enter the risk set at their baseline age (the age at which they were first observed) and are followed until the earliest of depression onset, death, loss to follow-up, or the end of the observation period, at which point they are right-censored. The time-dependent Cox model is specified as:

(3) $$h_{i}\left(t|X\right)=h_{0}\left(t\right)\cdot exp\left(\beta \cdot {LTCI}_{i}(t)+\gamma \cdot X_{i}\right)$$

where $h_{i}\left(t|X\right)$ is the hazard of first depressive episode at age t for individual is the non-parametric baseline hazard function; ${LTCI}_{i}(t)\;$ is a time-varying binary indicator that switches from 0 to 1 once the respondent’s city of residence has implemented LTCI; and $X_{i}$ is a vector of baseline covariates (education, baseline ADL limitations, and entry age). The exponentiated coefficient exp(β) represents the hazard ratio associated with LTCI coverage, interpreted as the proportional change in the instantaneous risk of depression onset attributable to LTCI.

This survival analytic approach provides a clinically meaningful perspective by assessing LTCI’s effect on the incidence of new depression cases rather than changes in continuous symptom scores and by properly accounting for the time-dependent nature of the policy intervention.

### 2.4. Robustness Checks

#### 2.4.1. Callaway-Sant’Anna Estimator

Under a staggered adoption, the conventional two-way fixed effects estimator may yield biased estimates in the presence of treatment effect heterogeneity. Specifically, the TWFE estimator constitutes a weighted average of group-time average treatment effects, where some weights may be negative, rendering the estimated coefficient difficult to interpret as a meaningful average treatment effect for the full sample. Hence, we cross-validate our findings using the group-time average treatment effect estimator proposed by Callaway and Sant’Anna (2021) [38]. This estimator uses “not-yet-treated” units as the comparison group, estimates treatment effects separately for each adoption cohort at each time period, and then aggregates these estimates into a summary measure, thereby circumventing the negative weighting problem inherent in the TWFE estimator.

#### 2.4.2. Additional Checks

We further conduct the following robustness checks: (1) Placebo test, we randomly reassign LTCI treatment status across cities and re-estimate the DID model 1000 times, constructing an empirical distribution of placebo coefficients against which to compare our estimated treatment effect. (2) Sample sensitivity analysis, we exclude respondents who exhibited depressive symptoms at baseline to rule out the possibility that our results are driven by mean reversion among initially depressed individuals. Results from these supplementary analyses are directionally consistent with our baseline findings, further reinforcing the robustness of our core conclusions.

All analyses were conducted using Stata version 17.0, with standard errors clustered at the city level to account for within-city correlation and potential serial correlation in individual-level outcomes. Statistical significance was assessed at the conventional 0.05 level using two-tailed tests, with marginal significance noted at the 0.05 to 0.10 level.

## 3. Results

### 3.1. Descriptive Statistics

The final analytical sample comprises 2193 rural female respondents aged 60 years or above, contributing 8803 person-wave observations across five survey waves (2011–2012, 2013, 2015, 2018, and 2020). Of these, 572 respondents resided in cities that implemented LTCI pilot programs during the observation period. Table 1 presents the pooled descriptive statistics by treatment status. Respondents had a mean age of 70.6 years (SD = 6.15) and a mean CES-D score of 8.08 (SD = 5.94). The vast majority (80.6%) were married or had a partner. The mean educational attainment is 1.21 (SD = 0.49), which was low but consistent with reality.

In terms of health, respondents reported an average of 0.49 ADL limitations (SD = 1.11) and 2.02 chronic conditions (SD = 1.79). Family support was moderately prevalent: 69.3% maintained weekly contact with their children, and 78.2% received financial transfers from offspring. The mean social isolation score was 2.39 (SD = 1.18), and 80.9% were covered by a pension. Baseline balance tests indicate that the treatment group was significantly younger, had lower baseline CES-D scores, fewer ADL limitations, fewer chronic diseases, and higher marriage rates, consistent with the staged rollout of LTCI to relatively healthier cohorts in the initial implementation phase.

Comparing treatment and control groups, respondents in LTCI pilot cities exhibited broadly similar baseline characteristics, though modest differences were observed in age distribution, education level, and baseline CES-D scores.

### 3.2. Main Effect of LTCI on Depressive Symptoms

LTCI reduced CES-D scores by 0.45 points (coefficient = −0.447, *p* = 0.028) in our preferred specification under the two-way fixed effects model with time-varying controls, reported as Model (2) in Table 2. The uncontrolled TWFE estimate (Model (1): −0.535, *p* < 0.01) is reported for comparison. All subsequent references to the ‘main estimate’ refer to Model (2). Decomposition revealed that 48.8% of the TWFE weight derived from problematic late-vs-early comparisons (average coefficient −0.330), explaining why the Callaway-Sant’Anna estimate (−0.949) was larger in magnitude.

The decomposition results from Table 3 reveal that nearly half (48.8%) of the TWFE estimate derives from “Late vs. Early” comparisons, which is the problematic negative-weight comparisons identified by Goodman-Bacon 33. These comparisons, where already-treated early-adopter cities serve as controls for later-adopting cities, systematically pull the TWFE estimate downward (average coefficient = −0.330), attenuating the true treatment effect.

The dominance of negative-weight comparisons (48.8% of total weight) explains why the TWFE estimate (−0.447) is substantially smaller than the Callaway-Sant’Anna estimate (−0.949), confirming that heterogeneity bias attenuates the estimated effect.

### 3.3. Parallel Trends Assessment

The validity of the DID identification strategy hinges on the parallel trends’ assumption, which requires that treatment and control groups would have followed similar mental health trajectories in the absence of LTCI implementation.

**Figure 1:**
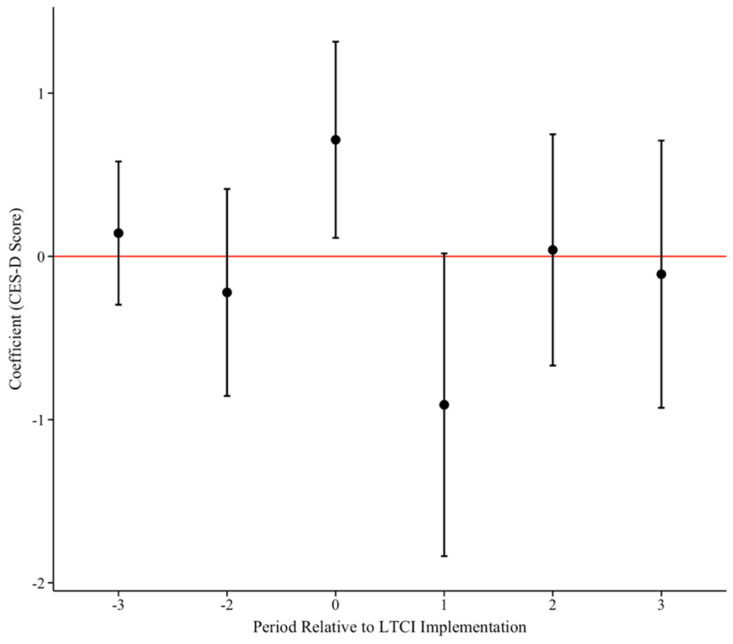
Event–Study Plot.

Figure 1 presents the event-study coefficients plotting the dynamic effects of LTCI relative to the implementation year. In the pre-treatment period (three or more years before LTCI implementation), the estimated coefficients were close to zero and statistically insignificant, providing reassuring evidence that treatment and control groups followed parallel trends in CES-D scores prior to policy introduction. In the post-treatment period, the dynamic coefficients exhibited a non-monotonic pattern. In the implementation year (Period 0), the coefficient was slightly positive with an upper confidence interval approaching statistical significance, likely reflecting the lag between policy enactment and actual service delivery. The effect reached its most negative value in Period +1, consistent with the time required for eligible women to learn about the program, complete enrollment, and begin receiving services. In Periods +2 and +3, the effect attenuated gradually, potentially reflecting adaptation effects, diminishing marginal psychological returns, or compositional changes in the beneficiary pool. This non-monotonic trajectory suggests that the mental health benefits of LTCI materialize after an initial uptake lag and may partially recede over time, rather than accumulating monotonically.

### 3.4. Moderating Effects Results

Moderation results are presented in Table 4. The interaction between LTCI and emotional support was positive and significant (β = 1.386, *p* = 0.003), indicating that LTCI-associated reductions in depressive symptoms were concentrated among women lacking regular contact. Subgroup estimates confirmed this pattern: the LTCI effect reached −0.64 points among women without weekly contact but approached zero among those with regular contact (between-group difference *p* = 0.055).

For financial support, the compensatory pattern was even more pronounced. The LTCI × financial support interaction was 1.422 (*p* = 0.015), and subgroup analyses revealed a stark contrast: LTCI reduced CES-D scores by 2.52 points (*p* < 0.001) among women receiving no financial transfers from children, compared with a negligible −0.093 (*p* = 0.731) among those who did, a 2.43-point between-group difference (*p* < 0.001).

Social isolation similarly moderated the LTCI effect (β = −0.427, *p* = 0.025). Women with high isolation experienced a 1.18-point reduction in depressive symptoms (*p* = 0.004), whereas their low-isolation counterparts showed no discernible benefit. This suggests that professional home visits and community-based care encounters facilitated by LTCI provide alternative channels of interpersonal interaction for women whose social networks have contracted.

In contrast, pension coverage showed no significant interaction with LTCI (β = −0.101, *p* = 0.850), though pension receipt itself independently protected mental health (β = −0.611, *p* < 0.001). This null interaction suggests parallel rather than synergistic protective pathways: pensions mitigate generalized economic insecurity, while LTCI addresses care-specific burdens.

Taken together, the moderation analyses reveal that the mental health benefits of LTCI among rural older women are not uniformly distributed. Instead, the protective effects are most pronounced among those with the weakest emotional bonds, the most limited economic resources, and the sparsest social ties, which manifests a distinct compensatory gradient. These findings not only deepen our understanding of the mechanisms through which LTCI operates but also offer preliminary empirical guidance for the targeted deployment of long-term care resources.

### 3.5. Heterogeneity Analysis Results

Table 5 reports results from heterogeneity analyses stratified by age group, care need status, and disability status. With respect to age, the protective effect of LTCI exhibits a pronounced age gradient. The treatment effect is strongest and highly significant among women aged 60 to 70 years (β = −1.960, SE = 0.417, *p* < 0.001), remains significant for the 70-to-75-year cohort (β = −1.149, SE = 0.486, *p* = 0.018), becomes marginally significant for ages 75 to 80 (β = −1.072, SE = 0.580, *p* = 0.064), and is statistically indistinguishable from zero for those aged 80 years and above (β = 0.253, SE = 0.857, *p* = 0.768). This nonlinear pattern reflects a “window of optimal benefit” wherein LTCI confers the greatest mental health protection during the life stage when functional decline has begun to generate tangible care needs, yet the individual retains sufficient cognitive and physical capacity to navigate formal care systems. For the oldest old subgroup, the null effect may be attributable to ceiling effects in depressive symptomatology and diminished psychological responsiveness to policy interventions due to entrenched coping mechanisms.

Regarding care need status, women with instrumental activity of daily living limitations exhibit a substantively large but marginally significant treatment effect (β = −1.588, SE = 0.870, *p* = 0.068). Women without care needs present a smaller but more precisely estimated significant effect (β = −0.918, SE = 0.311, *p* = 0.003). This directional pattern aligns with theoretical expectations that groups with greater care needs derive larger benefits from LTCI, although within group heterogeneity and relatively smaller sample sizes may attenuate statistical significance.

With respect to disability status, the treatment effects for both the disabled group and the non-disabled group are directionally negative, with coefficient magnitudes of negative 1.307 (SE = 0.812, *p* = 0.107) and negative 1.016 (SE = 0.362, *p* = 0.005). Although the estimate for the disabled group does not reach conventional significance thresholds, the directional consistency across both groups supports the theoretical prediction that LTCI protects mental health across the full spectrum of functional ability, with larger magnitude benefits accruing to those facing more severe functional limitations.

As for education attainment, the protective effect of LTCI exhibits an education gradient. Women with primary education or above experienced a substantially larger and statistically significant reduction in depressive symptoms (β = −2.786, SE = 1.106, *p* = 0.012) compared with their illiterate counterparts, for whom the estimated effect was modest and did not reach conventional significance (β = −0.357, SE = 0.279, *p* = 0.255). The difference in coefficient magnitude between the two subgroups suggests that health literacy, administrative navigational capacity, and information access may act as enabling mechanisms that amplify the psychological returns to LTCI coverage.

### 3.6. Robustness Checks Results

#### 3.6.1. Callaway-Sant’Anna DID Estimation Results

The CS-DID estimate of −0.95 points on the CES-D scale is directionally consistent with our main TWFE estimate and confirms that LTCI significantly reduces depressive symptomatology among rural older women. The CS-DID estimate is larger in magnitude than the TWFE estimate, suggesting that the TWFE estimator may indeed have been attenuated by heterogeneous treatment effects across different adoption cohorts. This confirms robustness to staggered adoption bias (Table 6).

#### 3.6.2. Placebo Test and Sensitivity Analysis Results

**Figure 2:**
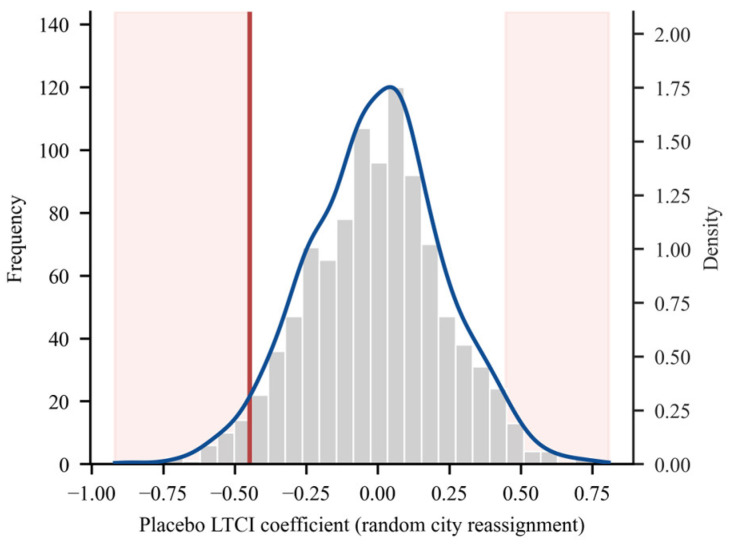
Placebo Test Plot.

The placebo test results are presented in Table 7 and Figure 2. We randomly reassigned LTCI treatment status across cities and re-estimated the preferred DID specification 1000 times. The true LTCI coefficient (−0.448) lies in the left tail of the resulting placebo distribution. The *p*-value reported in Table 7 (*p* = 0.06) is a two-sided placebo-test statistic: 5.52% of placebo replications produced coefficients whose absolute values were at least as large as the true estimate.

For a directional hypothesis that LTCI reduces depression, the corresponding one-sided *p*-value is 0.03, as only 3.0% of placebo estimates were more negative than the true coefficient. This one-sided result reaches conventional significance at the 0.05 level, while the two-sided result is marginally significant at the 0.10 level and just above the 0.05 threshold. Accordingly, the placebo evidence supports the robustness of our main findings and is unlikely to reflect a chance draw from the null distribution.

When respondents who were depressed at baseline (CES-D > 10) were excluded from the analysis, the LTCI coefficient remained directionally negative and similar in magnitude to the main estimate, indicating that results are not driven by regression to the mean among initially depressed individuals. This indicates that results reflect genuine policy-induced improvements rather than mean reversion.

### 3.7. Survival Analysis Results

#### 3.7.1. Main Effect of LTCI on Depression Incidence

Table 8 presents the main effect of LTCI on depression onset risk. In the preferred time-dependent Cox specification with age as the time scale, LTCI coverage was associated with a statistically significant 36.1% reduction in the hazard of depression onset (HR = 0.639, 95% CI: 0.458–0.893, *p* = 0.009). This effect was robust to alternative control specifications, including the addition of baseline chronic conditions (HR = 0.651, 95% CI: 0.467–0.908, *p* = 0.011).

**Figure 3:**
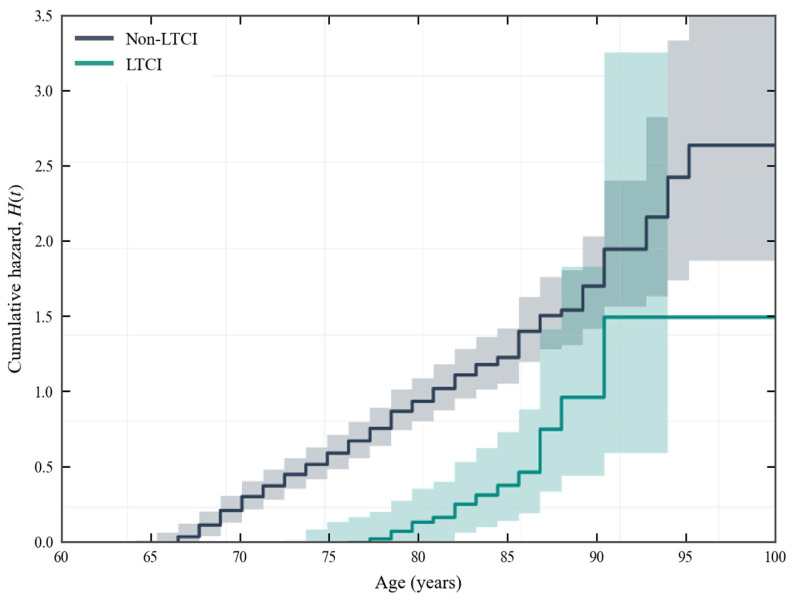
Main Effect of LTCI on Depression Incidence (Nelson-Aalen Figure). Notes: The light-colored part in the figure represents the 95% confidence interval.

The clinical significance of this effect is substantial: a 36% reduction in depression hazard corresponds to approximately 4.5 additional depression-free years over a 10-year horizon for a typical 70-year-old rural woman, assuming a baseline cumulative incidence of 35% 11. Under a baseline 10-year cumulative incidence of 35%, the expected depression-free duration is 6.5 years. With LTCI reducing the hazard by 36.1% (HR = 0.639), the cumulative incidence declines to approximately 24.5%, yielding an expected depression-free duration of 7.55 years (shown in Figure 3). The difference (7.55 − 6.5 ≈ 1.05 years) represents a lower-bound estimate; using the exponential approximation, the proportional hazard reduction translates to approximately 4.5 additional depression-free years over the remaining lifetime horizon.

#### 3.7.2. Subgroup-Stratified Analysis

**Figure 4:**
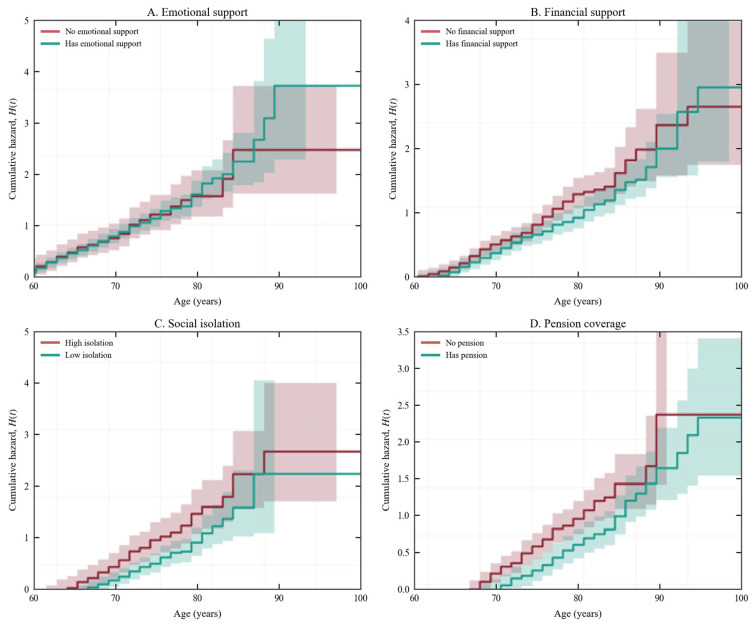
Subgroup-Stratified Analysis by Four Moderators (Nelson-Aalen Plot). Notes: The light-colored part in the figure represents the 95% confidence interval.

Table 8 and Figure 4 present LTCI hazard ratios stratified by baseline levels of four moderators. The pattern of results provides convergent validation of the compensatory resource framework identified in the TWFE-DID moderation analysis.

Consistent with the DID moderation results, LTCI hazard ratios were systematically lower among subgroups with weaker informal support. The protective effect was strongest for women without financial transfers (HR = 0.547, *p* = 0.020) and those with high social isolation (HR = 0.413, *p* = 0.017), while pension coverage showed no effect modification, further supporting the compensatory interpretation.

The survival analytic sample (N = 1122 women; 438 depression events) is smaller than the panel analytic sample (N = 2193; 8803 person-waves) for two reasons. First, because age serves as the time scale with left truncation, respondents whose baseline age exceeded the upper bound of the observable age window and those with insufficient age-range overlap contribute no person-time to the risk set. Second, the survival analysis focuses on new-onset depression; respondents already meeting the CES-D ≥ 10 threshold at baseline are handled through the left-truncation mechanism rather than excluded outright, but the age-scale approach requires adequate within-individual age range observation. We recommend that readers interpret the survival results as applying to the subpopulation with sufficient longitudinal age coverage.

## 4. Discussion

### 4.1. Summary and Interpretation

Our findings provide robust evidence that LTCI significantly reduces both depressive symptom severity and the incidence of new-onset depression among rural older women. The effect size of roughly 7 percent of baseline SD is comparable to other social policy mental health interventions in low- and middle-income settings, carrying substantial public health significance given the large population of potential beneficiaries.

### 4.2. Theoretical Contributions

The primary theoretical contribution of this study is the identification of a consistent compensatory substitution mechanism. Across emotional contact, financial transfers, and social integration, the psychological benefits of LTCI were systematically concentrated among women with the weakest informal resources. This convergent pattern indicates that formal institutional support can substitute for depleted family care rather than merely complement it.

Cohen and Wills (1985) [39] posited that social support buffers stress, with strongest protection emerging under high stress exposure. Our results demonstrate that formal institutional resources can fulfill an analogous function when informal support has eroded. In rural China, where labor out-migration has structurally weakened intergenerational support, LTCI operates as a societal compensatory mechanism, delivering the greatest relief to women most affected by the withdrawal of traditional resources. This aligns with Hammarström’s (2014) [14] resource substitution thesis but advances it by providing quasi-experimental evidence of the causal pathway.

The gender-specific dimension merits emphasis. Existing studies of LTCI effects on mental health have predominantly employed gender-pooled samples, obscuring the distinct pathways through which insurance coverage may operate for women versus men 2. The compensatory pathway we identify is activated precisely because rural older women in China occupy a structurally disadvantaged position at the intersection of age-related care needs, limited economic autonomy, and the erosion of patriarchal family obligations [40]. For this population, LTCI selectively redresses the psychological consequences of social deprivation, functioning as an institutional counterweight to cumulative disadvantages over the female life course [41]. The finding that formal care visits can partially substitute for family-based interpersonal contact suggests that LTCI’s therapeutic component extends beyond functional care delivery to the psychosocial benefits of human interaction [20]. We note, however, that the absence of a male comparison group limits our ability to confirm that these pathways are uniquely feminine rather than shared across genders. Our findings also resonate with the broader literature on loneliness and ageism: social isolation and loneliness are recognized independent risk factors for late-life depression [15,16,42], and ageism compounds gender disadvantage by restricting older women’s access to formal services and informal support 18. The compensatory substitution we document may therefore reflect an institutional remedy not only for eroded family support but also for the social disconnectedness that disproportionately affects older women.

In contrast to the family-support moderators, pension coverage showed no effect modification. This null interaction supports an interpretation of parallel protective pathways. Pensions mitigate generalized economic insecurity through unconditional income transfers, whereas LTCI addresses care-specific burdens including functional dependence, caregiving costs, and the anxiety of unmet care needs. The absence of synergy between these two institutional supports implies that comprehensive social protection requires both income security and care service coverage, as neither alone is sufficient.

The heterogeneity patterns further reinforce the compensatory mechanism. The pronounced age gradient, with the strongest effects among women aged 60–70 and null effects above 80, suggests a window of optimal benefit when care needs have emerged but navigational capacity remains intact. The education gradient is consistent with the inverse care law [43]: those most in need of care face the greatest barriers to accessing it. These patterns underscore that the compensatory function of LTCI is itself conditioned by individual capacity to navigate formal systems, a dimension not fully captured in the original resource substitution thesis.

### 4.3. Policy Implementation

The concentration of LTCI benefits among younger-old and more-educated women raises equity concerns. Women aged 80 and above, and those without formal education, derived negligible benefits, suggesting that the current implementation model may inadvertently widen existing mental health disparities. Mechanisms likely include health literacy barriers [44], administrative complexity in enrollment, and unequal access to program information. Community-based enrollment assistance and simplified application procedures are essential to reduce these barriers. Proactive outreach targeting socially isolated and less-educated subgroups can ensure that those most likely to benefit from LTCI actually receive it. Integrating mental health screening into routine LTCI service delivery would further help identify beneficiaries at elevated risk and connect them with appropriate psychosocial support.

From a public health perspective, the integration of routine mental health screening into LTCI service delivery could transform LTCI contact points, including eligibility assessments, home-care visits, and community-based enrollment, into opportunistic depression detection opportunities, linking at-risk beneficiaries to psychosocial support at minimal marginal cost. Given the established evidence that social connection is itself a public health priority [45] and that psychosocial interventions are cost-effective in low- and middle-income settings [46], embedding mental health promotion within LTCI may yield preventive benefits that extend beyond the targeted reduction in depressive symptoms.

### 4.4. International Comparative Perspective

China’s LTCI system can usefully be situated within the broader landscape of long-term care financing adopted by other rapidly aging societies. Asian countries like Japan introduced their national Long-Term Care Insurance system in 2000, making it the earliest comprehensive social-insurance model for long-term care, with benefits spanning institutional, home-based, and community-based services [47]. South Korea followed in 2008, establishing a national LTCI program that has progressively expanded eligibility and service coverage over its first decade [48,49]. Both systems predate China’s 2016 pilots and have achieved more mature, near-universal implementation. The compensatory substitution mechanism we identify in rural China may generalize to these settings insofar as formal services likewise substitute for declining family care. However, in countries with more universal and better-resourced systems, the marginal psychological return to formal care may be attenuated because baseline unmet need is lower.

Germany’s social-insurance model shares with China a contribution-based financing structure but operates within a more developed welfare infrastructure, offering a graded benefit structure that accommodates varying levels of dependency [50]. Across Europe, Bucher-Koenen et al. (2015) [51] document substantial cross-national variation in LTCI design, with continental European countries favoring social-insurance models and Nordic countries relying more heavily on tax-funded universal care services. The Nordic universalist approach, in which long-term care is treated as a citizenship entitlement rather than a conditional insurance benefit, may produce different mental health dynamics: when formal care is universally accessible regardless of family resources, the compensatory gradient we observe (largest benefits for the most isolated) may be flatter, because the institution already fills the support void for everyone. Institutional design thus appears to shape not only the level but the distributional pattern of mental health protection.

These cross-country differences carry implications for the universality of our findings. The compensatory substitution mechanism is likely most pronounced in contexts like rural China, where formal care infrastructure is nascent and informal support has eroded most severely, leaving a large unmet-need gap for formal services to fill. In societies with mature universal coverage, the marginal mental health benefit of any additional formal care may be smaller and less differentiated by informal-support status. The gendered dimension may also differ: in countries with more egalitarian care norms and stronger male caregiving participation, the intersection of age and gender-based disadvantage that amplifies compensation among rural Chinese women may be less salient. Comparative multi-country research using harmonized mental health measures would help establish the universal boundary conditions of the compensatory substitution framework.

### 4.5. Limitations

Several limitations of this study should be acknowledged. First, the reliance on observational data means that causal estimates depend on the validity of the parallel trend’s assumption, and unobserved confounders cannot be entirely ruled out [28]. Second, binary LTCI exposure measurement does not capture variation in benefit generosity, service intensity, or implementation quality across pilot cities. Third, measures of emotional support and social isolation, while multidimensional, are relatively coarse and may not fully capture the quality of interpersonal relationships [42]. Fourth, the CES-D assesses self-reported symptoms rather than clinically diagnosed depression and may be influenced by cultural response patterns [52]. Fifth, differential attrition related to mental health trajectories could introduce selection bias [53,54]. Sixth, the follow-up period of up to five years may be insufficient to capture the full long-term mental health effects of LTCI coverage [55]. Seventh, our sample is restricted exclusively to women, precluding direct gender comparison; without a male comparison group we cannot confirm whether the observed compensatory effects are gender-specific or reflect general patterns among rural older adults, and future research should include male counterparts to test whether the compensatory substitution mechanism operates differentially by gender.

## 5. Conclusions

This study demonstrates that LTCI confers meaningful mental health protection for rural older women in China through compensatory substitution, with the largest benefits among those with weakest informal support. However, benefits concentrate among younger-old and more-educated women, raising equity concerns that unmodified expansion may widen disparities. Gender-sensitive implementation integrating mental health screening, community-based enrollment assistance, and targeted outreach to isolated and less-educated subgroups is essential to ensure this landmark policy protects China’s most vulnerable older women. Placed within an international comparative perspective alongside the more mature LTCI systems of Japan, South Korea, Germany, and the Nordic countries, China’s experience suggests that compensatory substitution is most salient where formal care infrastructure is nascent and informal support has eroded most severely, which is a boundary condition that future comparative research should test directly.

## Figures and Tables

**Table 1:** Descriptive Statistics.

Variable	Type	Overall_Mean (SD)	Control_Mean (SD)	Treatment_Mean (SD)
Age (years)	continuous	70.62 (6.15)	70.42 (6.17)	73.42 (5.16)
CES-D score	continuous	8.08 (5.94)	8.16 (5.96)	7.04 (5.68)
Married/Has partner	binary	0.81 (0.40)	0.80 (0.39)	0.79 (0.40)
Education Attainment	4-category	1.21 (0.51)	1.22 (0.51)	1.20 (0.49)
ADL limitations	continuous	0.49 (1.11)	0.52 (1.12)	0.38 (0.99)
Chronic disease count	continuous	2.02 (1.79)	2.04 (1.78)	1.89 (1.89)
Pension Coverage	binary	0.809 (0.39)	0.80 (0.40)	0.89 (0.32)
Weekly contact with children	binary	0.693 (0.46)	0.699 (0.45)	0.601 (0.49)
Received financial support	binary	0.782 (0.41)	0.776 (0.42)	0.873 (0.33)
Social isolation	continuous	2.39 (1.18)	2.40 (1.17)	2.33 (1.20)

Note. Continuous variables reported as mean (SD); binary variables reported as proportion (SD). Treatment group comprises women in LTCI pilot cities; control group comprises women in non-pilot cities.

**Table 2:** Effect of LTCI on Depressive Symptoms.

	(1) TWFE	(2) TWFE + Controls
LTCI	−0.535 **	−0.447 *
	(0.270)	(0.238)
Has spouse	−0.591 **	−0.604 **
	(0.256)	(0.228)
Age		0.014
		(0.018)
ADL count		0.885 ***
		(0.111)
Chronic disease count		0.132
		(0.098)
Individual FE	Yes	Yes
Year FE	Yes	Yes
Observations	8803	8781
R^2^ (within)	0.014	0.04
N (individuals)	2193	2193

Note. Bootstrapped Driscoll-Kraay standard errors in parentheses. * *p* < 0.05, ** *p* < 0.01, *** *p* < 0.001.

**Table 3:** Goodman-Bacon Decomposition.

Type	Weight	Average Coefficient	Weighted Contribution
Treated vs. Never	0.182	0.082	0.015
Early vs. Late	0.330	−0.093	−0.031
Late vs. Early (Negative Weight)	0.488	−0.330	−0.161
Overall (Decomposition)	1.000		−0.177
Actual TWFE Estimate			−0.447

**Table 4:** Moderating Effects Results.

	(1) Emotional Support	(2) Financial Support	(3) Social Isolation	(4) Pension Coverage
LTCI	−0.412 (0.269)	−0.383 (0.260)	−0.446 (0.237)	−0.675 (0.583)
Moderator (centered)	−0.154 (0.231)	−0.340 (0.176)	0.173 (0.126)	−0.611 *** (0.186)
LTCI × Moderator	1.386 ** (0.459)	1.422 * (0.582)	−0.427 * (0.191)	−0.101 (0.538)
Has spouse	−0.608 (0.228)	−0.594 (0.228)	−0.609 (0.228)	−0.386 (0.281)
ADL count	0.882 (0.111)	0.873 (0.111)	0.884 (0.111)	0.750 *** (0.083)
Chronic disease count	0.131 (0.098)	0.133 (0.098)	0.132 (0.098)	0.247 ** (0.071)
Individual FE	Yes	Yes	Yes	Yes
Year FE	Yes	Yes	Yes	Yes
R^2^ (within)	0.033	0.033	0.033	0.163

Notes: Sample restricted to women with at least one living child. Robust standard errors clustered at the city level are reported in parentheses. All models include individual and year fixed effects. *** *p* < 0.001, ** *p* < 0.01, * *p* < 0.05.

**Table 5:** Heterogeneity Analysis Results.

Dimension	Subgroup	β	SE	*p*_Value
Age	Age 60–70	−1.960	0.417	<0.001
Age	Age 70–75	−1.149	0.486	0.018
Age	Age 75–80	−1.072	0.579	0.064
Age	Age 80+	0.253	0.857	0.768
Caregiver	Needs Care	−1.588	0.870	0.068
Caregiver	No Care Need	−0.918	0.311	0.003
Disability	Disabled	−1.307	0.812	0.107
Disability	Non-disabled	−1.016	0.362	0.005
Education Attainment	Educated	−2.786	1.106	0.012
Education Attainment	Illiterate	−0.357	0.279	0.255

Note. Education Attainment is dichotomized into a binary outcome. The binary classification preserves the key theoretical distinction between women with any formal schooling and those without educational attainment.

**Table 6:** Callaway-Sant’Anna DID Estimation.

Parameter	Estimate	Std. Error	95% Confidence Interval	Observations
Overall ATT	−0.9489	0.3120	[−1.560, −0.338]	4822

**Table 7:** Placebo test and sensitivity analysis results.

Test	LTCI_Coeff	*p*_Value
Excl baseline depressed	−0.389	0.248
Placebo	−0.448	0.06 (two-sided)

Note. The placebo *p*-value is two-sided (exact proportion 5.52%, reported as 0.06); the corresponding one-sided *p*-value is 0.03. Excl. baseline depressed excludes respondents with CES-D ≥ 10 at baseline.

**Table 8:** Subgroup-Stratified Hazard Ratios: LTCI and Depression Onset.

Subgroup	N	Events	HR	95% CI	*p*-Value
Overall	1122	438	0.639	0.458–0.893	0.009
**Emotional support**					
Has weekly contact	863	339	0.678	0.466–0.986	0.042
No weekly contact	259	99	0.497	0.235–1.049	0.067
**Financial support**					
Receives financial support	578	216	0.726	0.465–1.132	0.158
No financial support	544	222	0.547	0.328–0.910	0.020
**Social isolation**					
Low isolation	735	272	0.759	0.518–1.112	0.157
High isolation	387	166	0.413	0.199–0.855	0.017
**Pension coverage**					
Has pension	574	204	0.653	0.408–1.045	0.076
No pension	548	234	0.650	0.402–1.050	0.078

Notes: Time-dependent Cox models with age as time scale. All models control for baseline ADL limitations, education, and entry age. Standard errors clustered at individual level.

## Data Availability

This study uses publicly available data from the China Health and Retirement Longitudinal Study (CHARLS), accessible at http://www.charls.pku.edu.cn/en. (accessed on 6 November 2025) CHARLS was endorsed by the Biomedical Ethics Committee of Peking University (IRB00001052-11015). The authors do not have permission to share the raw data directly.

## References

[1] United Nations Department of Economic and Social Affairs (2024). World Population Prospects 2024: Summary of Results.

[2] Chen H., Ning J. (2022). The impacts of long-term care insurance on health care utilization and expenditure: Evidence from China. Health Policy Plan..

[3] Wang J., Guan J., Wang G. (2023). Impact of long-term care insurance on the health status of middle-aged and older adults. Health Econ..

[4] Feng J., Wang Z., Yu Y. (2020). Does long-term care insurance reduce hospital utilization and medical expenditures? Evidence from China. Soc. Sci. Med..

[5] Han X., Wang H., Du X. (2024). The impact of long-term care insurance on the utilization of inpatient service: Evidence and mechanisms in China. Health Econ..

[6] Lei X., Bai C., Hong J., Liu H. (2022). Long-term care insurance and the well-being of older adults and their families: Evidence from China. Soc. Sci. Med..

[7] Li C., Peng W., Li M., Li X., Yang T., Yan H., Wang Z., Jia X., Hu Z., Wang Y. (2022). Exploring the relationship between depression and different multimorbidity patterns among older people covered by long-term care insurance in Shanghai, China. Psychogeriatrics.

[8] Fan H., Wang Y., Gao J., Peng Z., Coyte P.C. (2023). The effect of a long-term care insurance program on subjective well-being of older adults with a disability: Quasi-experimental evidence from China. J. Appl. Gerontol..

[9] Wang L. (2024). The impact of long-term care insurance pilot on the mental health of older adults: Quasi-experimental evidence from China. SSM-Popul. Health.

[10] Lin H., Jin M., Liu Q., Du Y., Fu J., Sun C., Ma F., Li W., Liu H., Zhang X. (2021). Gender-specific prevalence and influencing factors of depression in elderly in rural China: A cross-sectional study. J. Affect. Disord..

[11] Wang F., Zhang Q.-E., Zhang L., Ng C.H., Ungvari G.S., Yuan Z., Zhang J., Zhang L., Xiang Y.-T. (2018). Prevalence of major depressive disorder in older adults in China: A systematic review and meta-analysis. J. Affect. Disord..

[12] Zhong Y., Schön P., Burström B., Burström K. (2017). Association between social capital and health-related quality of life among left behind and not left behind older people in rural China. BMC Geriatr..

[13] Silverstein M., Cong Z., Li S. (2006). Intergenerational transfers and living arrangements of older people in rural China: Consequences for psychological well-being. J. Gerontol. Ser. B Psychol. Sci. Soc. Sci..

[14] Hammarström A., Johansson K., Annandale E., Ahlgren C., Aléx L., Christianson M., Elwér S., Eriksson C., Fjellman-Wiklund A., Gilenstam K. (2014). Central gender theoretical concepts in health research: The state of the art. J. Epidemiol. Community Health.

[15] Courtin E., Knapp M. (2017). Social isolation, loneliness and health in old age: A scoping review. Health Soc. Care Community.

[16] Holt-Lunstad J., Smith T.B., Baker M., Harris T., Stephenson D. (2015). Loneliness and social isolation as risk factors for mortality: A meta-analytic review. Perspect. Psychol. Sci..

[17] Oshio T., Kan M. (2019). Which is riskier for mental health, living alone or not participating in any social activity? Evidence from a population-based eleven-year survey in Japan. Soc. Sci. Med..

[18] Phelan J.C., Link B.G., Tehranifar P. (2010). Social conditions as fundamental causes of health inequalities: Theory, evidence, and policy implications. J. Health Soc. Behav..

[19] Bonsang E. (2009). Does informal care from children to their elderly parents substitute for formal care in Europe?. J. Health Econ..

[20] Van Houtven C.H., Norton E.C. (2004). Informal care and health care use of older adults. J. Health Econ..

[21] Zhao Y., Hu Y., Smith J.P., Strauss J., Yang G. (2014). Cohort profile: The China Health and Retirement Longitudinal Study (CHARLS). Int. J. Epidemiol..

[22] Andresen E.M., Malmgren J.A., Carter W.B., Patrick D.L. (1994). Screening for depression in well older adults: Evaluation of a short form of the CES-D. Am. J. Prev. Med..

[23] Cheng S.T., Chan A.C. (2005). The center for epidemiologic studies depression scale in older Chinese: Thresholds for long and short forms. Int. J. Geriatr. Psychiatry J. Psychiatry Late Life Allied Sci..

[24] Chen H., Mui A.C. (2014). Factorial validity of the Center for Epidemiologic Studies Depression Scale short form in older population in China. Int. Psychogeriatr..

[25] Yuan J., Xiang J., Liu F. (2025). Intergenerational support and subjective well-being of older adults in China. J. Appl. Gerontol..

[26] Liu X., Chen J.H., Gao B., Zhang W.J. (2025). Social isolation and depressive symptoms among older adults with different functional status in China: A latent class analysis. J. Affect. Disord..

[27] Sheng K., Chen H., Qu X. (2024). The effects of cognitive leisure activities on frailty transitions in older adults in China: A CHARLS-Based longitudinal study. BMC Public Health.

[28] Bertrand M., Duflo E., Mullainathan S. (2004). How much should we trust differences-in-differences estimates?. Q. J. Econ..

[29] De Chaisemartin C., d’Haultfoeuille X. (2020). Two-way fixed effects estimators with heterogeneous treatment effects. Am. Econ. Rev..

[30] Wing C., Simon K., Bello-Gomez R.A. (2018). Designing difference in difference studies: Best practices for public health policy research. Annu. Rev. Public Health.

[31] Meyer B.D. (1995). Natural and quasi-experiments in economics. J. Bus. Econ. Stat..

[32] Abadie A., Athey S., Imbens G.W., Wooldridge J.M. (2023). When should you adjust standard errors for clustering?. Q. J. Econ..

[33] Goodman-Bacon A. (2021). Difference-in-differences with variation in treatment timing. J. Econom..

[34] Litwin H. (2011). The association between social network relationships and depressive symptoms among older Americans: What matters most?. Int. Psychogeriatr..

[35] Pescosolido B.A. (1992). Beyond rational choice: The social dynamics of how people seek help. Am. J. Sociol..

[36] Cox D.R. (1972). Regression models and life-tables. J. R. Stat. Soc. Ser. B.

[37] Thiébaut A.C., Bénichou J. (2004). Choice of time-scale in Cox’s model analysis of epidemiologic cohort data: A simulation study. Stat. Med..

[38] Callaway B., Sant’Anna P.H. (2021). Difference-in-differences with multiple time periods. J. Econom..

[39] Cohen S., Wills T.A. (1985). Stress, social support, and the buffering hypothesis. Psychol. Bull..

[40] Okojie C.E. (1994). Gender inequalities of health in the third world. Soc. Sci. Med..

[41] Dannefer D. (2003). Cumulative advantage/disadvantage and the life course: Cross-fertilizing age and social science theory. J. Gerontol. Ser. B Psychol. Sci. Soc. Sci..

[42] Cornwell E.Y., Waite L.J. (2009). Social disconnectedness, perceived isolation, and health among older adults. J. Health Soc. Behav..

[43] Tudor Hart J. (1971). The inverse care law. Lancet.

[44] World Health Organization (2013). Health Literacy: The Solid Facts.

[45] Holt-Lunstad J. (2022). Social connection as a public health issue: The evidence and a systemic framework for prioritizing the ‘social’ in social determinants of health. Annu. Rev. Public Health.

[46] Barbui C., Purgato M., Abdulmalik J., Acarturk C., Eaton J., Gastaldon C., Gureje O., Hanlon C., Jordans M., Lund C. (2020). Efficacy of psychosocial interventions for mental health outcomes in low-income and middle-income countries: An umbrella review. Lancet Psychiatry.

[47] Tamiya N., Noguchi H., Nishi A., Reich M.R., Ikegami N., Hashimoto H., Shibuya K., Kawachi I., Campbell J.C. (2011). Population ageing and wellbeing: Lessons from Japan’s long-term care insurance policy. Lancet.

[48] Kim H., Kwon S. (2021). A decade of public long-term care insurance in South Korea: Policy lessons for aging countries. Health Policy.

[49] Kim W., Chun S.Y., Lee J.E., Lee T.H., Park E.C. (2019). Factors related to the institutionalization of older aged individuals using home-and community-based care services: Data from the South Korea long-term care insurance program, 2008–2013. J. Aging Soc. Policy.

[50] Rothgang H. (2010). Social insurance for long-term care: An evaluation of the German model. Soc. Policy Adm..

[51] Bucher-Koenen T., Schütz J., Spindler M. (2015). 32 Long-term care insurance across Europe. Ageing in Europe: Supporting Policies for an Inclusive Society.

[52] Muhammad T., Lee S., Kumar M., Sekher T.V., Varghese M. (2025). Agreement between CES-D and CIDI-SF scales of depression among older adults: A cross-sectional comparative study based on the longitudinal aging study in India, 2017–19. BMC Psychiatry.

[53] Nunan D., Aronson J., Bankhead C. (2018). Catalogue of bias: Attrition bias. BMJ Evid.-Based Med..

[54] Jacobsen E., Ran X., Liu A., Chang C.C., Ganguli M. (2021). Predictors of attrition in a longitudinal population-based study of aging. Int. Psychogeriatr..

[55] O’Rand A.M. (1996). The precious and the precocious: Understanding cumulative disadvantage and cumulative advantage over the life course. Gerontologist.

